# ‘Make the Most of the Situation’. Older Adults’ Experiences during COVID-19: A Longitudinal, Qualitative Study

**DOI:** 10.1177/07334648221105062

**Published:** 2022-10

**Authors:** Emily Brooks, Somayyeh Mohammadi, W. Ben Mortenson, Catherine L. Backman, Chihori Tsukura, Isabelle Rash, Janice Chan, William C. Miller

**Affiliations:** 1 Department of Occupational Science and Occupational Therapy, Faculty of Medicine, 8166 University of British Columbia, Vancouver, BC, Canada; 2 Rehabilitation Research Program, GF Strong Rehabilitation Centre, Vancouver, BC, Canada; 3 Department of Psychology, 4264 Kingston University, London, UK; 4 8166 International Collaboration on Repair Discoveries, Vancouver, BC, Canada; 5 Graduate Program in Rehabilitation Sciences, Faculty of Medicine, 8166 University of British Columbia, Vancouver, BC, Canada

**Keywords:** COVID-19, transitions, well-being, social support, health

## Abstract

The COVID-19 pandemic restrictions have been associated with increased social isolation and reduced participation in older adults. This longitudinal qualitative study drew on life course theory to analyse data from a series of four sequential semi-structured interviews conducted between May 2020–February 2021 with adults aged 65+ (*n* = 12) to explore older adults’ experiences adjusting to the COVID-19 pandemic. We identified three themes: (1) *Struggling* ‘You realize how much you lost’ describes how older adults lost freedoms, social connections and activities; (2) *Adapting* ‘whatever happens, happens, I’ll do my best’, revealing how older adults tried to maintain well-being, participation and connection; and (3) *Appreciating* ‘enjoy what you have’, exploring how older adults found pleasure and contentment. Engagement in meaningful activities and high-quality social interactions supported well-being during the COVID-19 pandemic for older adults. This finding highlights the need for policies and services to promote engagement during longstanding global crises.

**What this paper adds**

• Point 1: Older adults strategically engaged in activities to promote their well-being during the COVID-19 pandemic, such as pursuing personal interests, remaining physically active and fostering high-quality relationships.
• Point 2: Older adults’ comfort in using community spaces was reduced when COVID-19 pandemic measures were suggested rather than mandated.
• Point 3: Adequate digital literacy, receiving support to learn new technologies and user-friendly websites improved older adults’ ability to access community services during the COVID-19 pandemic.

**Applications of study findings**

• Point 1: The government should develop publicly funded programs that provide opportunities for older adults to pursue personal interests, foster high-quality relationships and remain physically active.
• Point 2: Public emergency measures, such as COVID-19 pandemic restrictions, should be mandated rather than suggested in public spaces to increase older adults’ engagement in community activities.
• Point 3: Health providers should invest in user-friendly websites and other user-friendly communication modalities such as the telephone or in-person services for older adults.

## Introduction

In March 2020, the government of British Columbia (BC) instituted an evolving series of measures to reduce the spread of SARS-CoV-2. Pandemic restrictions may disproportionately impact the well-being of older adults (Miller, 2020). Because older adults face higher rates of mortality, complications, or comorbidities from SARS-CoV-2, they may adopt distancing measures more stringently than younger individuals to mitigate risk (Kivi et al., 2021). Furthermore, older adults are more likely to live alone, have lost family and friends, not be able to drive and be retired (Smith et al., 2020). Older adults reported increased social isolation and reduced participation in societal roles such as caregiving, resulting in declines in physical and mental well-being during the COVID-19 pandemic (Grotz et al., 2020; Miller, 2020). Pandemic restrictions also impacted older adults’ health by reducing their ability to remain active, obtain essential medical items and attend medical appointments (Visser et al., 2020).

In response to these challenges, studies reveal that some older adults employed strategies such as remaining active, learning new technologies and staying busy during the COVID-19 pandemic (Fiocco et al., 2021; Huntley & Bratt, 2022). A study by Fiocco et al. (2021) also indicated that older adults’ experiences during the COVID-19 pandemic were improved by social support, spacious or shared living arrangements and financial stability. This finding highlights the need for further exploration of how older adults adjusted to the COVID-19 pandemic and factors that influenced this adjustment, in order to guide institutions supporting older adults in sustaining activities contributing to their health and well-being.

To explore older adults’ experiences of adjusting to the COVID-19 pandemic, this paper adopts a life course perspective, which concerns changes in behaviour, health and resource accumulation over time, known as trajectories, as well as changes in roles or responsibility, known as transitions (Bernardi et al., 2019; Elder, 1995). These transitions and trajectories arise from the dynamic relationship among individual development (including personal experiences, health and resource accumulation), with culture and context (including social networks, institutions, and social and geographical locations). For example, someone with limited social networks and limited means has a different experience of retirement than someone who is more affluent and has extensive social networks. In both instances, personal experiences can shape transitions. The COVID-19 pandemic necessitated people of all generations to undergo transition. However, older adults have undergone numerous other life transitions prior to the pandemic, such as retirement, enabling them to draw on lessons from earlier experiences (Jonsson et al., 2001).

This study employed a life course perspective by exploring changes in roles and responsibilities (transitions) as well as health, behaviour and resources (trajectories), while considering context and individual development. This study aimed to explore older adults’ experiences of adjusting to the COVID-19 pandemic over a 10-month period to guide institutions to facilitate older adults’ engagement in activities essential for health, well-being and quality of life.

## Method

### Research Design

This 10-month longitudinal qualitative study drew on data from a larger project on the experiences of various populations (families, people with disabilities) during the pandemic (Reid et al., 2021). The local ethics board approved the study. Results are reported using the Consolidated Criteria for Reporting Qualitative Research (Tong et al., 2007).

### Participants and Recruitment

Participants were aged 65 and over; lived in Canada; had access to the internet and either a computer, smartphone or tablet; and were able to communicate through written and spoken English. The team recruited participants through advertisements on social media platforms, online postings and contacting previous participants at the research team’s centre. Eligible participants signed the consent form electronically before the first interview.

### Data Collection

Via video call using the platform Zoom, interviewers conducted four sequential semi-structured interviews of 45–60 minutes, with each interview taking place at a different timepoint (T1, T2, T3 and T4). T1 interviews were conducted between May and June 2020, during the first BC lockdown, the T2 interviews were conducted between June and July 2020, when socializing in bubbles of six was permitted and select non-essential services reopened, the T3 interviews were conducted between August and September 2020, when non-essential travel resumed, and T4 interviews were conducted between January and February 2021, after the initiation of the vaccine roll out (Covid-19 situation report, Week 17, 2021). Interviewers locked the interview rooms after participants entered to protect their privacy. Interview questions, based on Hammell’s (2009) classification of occupations, explored activities to connect and contribute, for restoration, and to connect the past to the future. Appendix 1 displays the T1 interview questions. The research team revised the interview guides in each timepoint to reflect changes in provincial guidelines. Due to the longitudinal nature of this study, participants were only recruited from May and June 2020 when the first round of interviews was conducted. No new participants were recruited after this timepoint.

### Analysis

Interviews were audio recorded, transcribed verbatim and anonymized. Coding was influenced by reflective thematic analysis outlined in Braun and Clarke (2006; 2021) in that the research team’s values, knowledge, and experiences mediated the generation of data into codes, and codes into themes. Additionally, coders familiarized themselves with the data prior to coding (Braun & Clarke, 2006; 2021). However, diverging from reflective thematic analysis, for pragmatic reasons, including co-ordination between numerous team members, codes were compiled into a codebook (Braun & Clarke, 2021). The use of the codebook is more structured which improved efficiency and communication between coders but restricted their ability to modify existing codes between timepoints relative to thematic analysis outlined by Braun and Clarke (2021). For the T1 interviews, coders coded the first three transcripts separately to explore varying interpretations of the data, before collaborating to co-construct codes. Conflicting codes were retained to honour varying constructions. To increase the efficiency of coding, the remaining transcripts were coded separately. Coders compiled the codes into initial codebooks which were then reviewed and edited by the research team. Coders used this initial codebook to analyse subsequent interviews (T2–T4). For T2–T4 interviews, the coding team coded the transcripts separately before meeting to discuss new codes or ambiguous cases, revisiting the original data if needed. The codebook was modified to include new codes that arose and reflect coders’ deepening conceptualizations of the data. These modifications were reviewed with the research team. Variations in the codes in each codebook were compared across interviews (T1–T4). Codes with conceptual similarity were grouped into categories by each researcher. Subsequently the team met to compare categories and develop themes. Life course perspective was implemented in the analysis phase, subsequent to coding data to guide development of themes. Life course perspective was selected as it aligned with the codes generated and provided a framework to explore the experiences of older adults during the COVID-19 pandemic deeply and comprehensively (Bernardi et al., 2019). One coder compiled a list of quotes from the categories for each theme, before converging on one code to represent each theme. The research team provided feedback on this selection.

### Trustworthiness Strategies

The team employed investigator triangulation by having two interviewers code the same data separately for the first three transcripts before meeting to discuss codes and provide rich, complementary perspectives (Salkind, 2010). The research team employed reflectivity by considering how their values, knowledge and skills mediated the data collection and generation of themes (Korstjens & Moser, 2018). The team consisted of four researchers with PhDs (SM, BM, WCM, CB), three of whom were registered as occupational therapists (BM, WCM, CB), one with a master’s degree working as an occupational therapist (EB), one PhD student (IR) and two Masters of Occupational Therapy Students (CT, JC). The coding team consisted of SM and EB both females in their 30s, and the interview team CT and EB, females in their 20s and 30s, respectively. All of research team were affiliated with the University of British Columbia. The interviewers and coders were volunteers. Interviewers recorded field notes detailing the content of the interview observations and power dynamics which includes specific instances where interviewers answered their own questions, or participants providing truncated responses or avoided question. Interviewers reviewed interview notes prior to subsequent interviews, thereby guiding the data collection process.

## Results

### Demographics

There were 12 participants in total which are displayed in Table 1. All participants completed T1–T4 interviews, except for one that did not complete T4. Seven participants were female. All participants lived in the community and the mean age was 72.83. All participants were retired, but two did intermittent contract work. Half of the participants lived alone and identified as Canadian. Of the participants who specified their income, all earned over CAD $14,999 per year, two earned CAD $14,999–$44,999, three earned CAD $45,000–$74,999 per year and four earned over CAD $75,000 per year. Of those who specified their level of education, 40% graduated college or university, 30% percent graduated post graduate education and 10% graduated high school or trade school.

**Table 1.** Demographics of Older Adults.

Name	Age	Sex	Live Alone
Frank	73	Male	no
Rose	76	Female	no
Eric	75	Male	no
Margret	65	Female	yes
Sue	73	Female	yes
Anne	80	Female	yes
Sarah	70	Female	yes
Tom	73	Male	yes
Alice	68	Female	no
Lily	73	Female	yes
Fred	76	Male	no
Terry	72	Male	no

### Themes

Our team identified three themes and eight subthemes displayed in Table 2: (1) *Struggling* ‘you realize how much you lost’, which including subtheme missing in-person engagements, burdens of risk and restrictions and declines in well-being; (2) *Adapting* ‘whatever happens, happens, I’ll do my best’, which includes the subthemes supporting well-being and sustaining connections; (3) A*ppreciating* ‘enjoy what you have’, which includes the subthemes life circumstance and experientially enjoying activities.

**Table 2.** Themes and Subthemes.

Struggling: ‘You realize how much you lost’. (Rose, T1)	
Burdens of risk and restrictions	‘You have to think before you do anything’ (Frank, T1)
Missing in-person engagements	‘It still bugs me that I can’t get together with my friends’ (Lily, T4)
Declines in health and well-being	‘All of a sudden, my age really caught up with me’ (Anne, T4)
Adapting: ‘Whatever happens, happens. I’ll do my best’. (Margret, T2)	
Supporting well-being	‘Walking was really important for my mental health’ (Alice, T3)
Sustaining connections	‘I find people are reconnecting [by email] more than they would have done before’ (Rose, T2)
Appreciating: ‘Enjoy what you have’. (Frank, T3).	
Life circumstances	‘I’m retired, I have a pension, we live in a beautiful place…I have a decent relationship’ (Alice, T3)
Experientially enjoying activities	‘Those are meaningful moments to me when I’m observing nature’ (Anne, T2)

#### *Theme 1: Struggling: ‘You realize how much you lost*’ *(Rose, T1)*

This theme explains participants’ struggles with the loss of in-person interactions and community activities and the adverse impacts of the pandemic on the participants’ health and well-being. This theme comprises of the subthemes: burdens of risk and restrictions, missing in-person engagements and declines in well-being.

*Burdens of risk and restrictions:* ‘I can get anxious when I go to shops’ (Rose, T1). In T1, all participants found community outings to be less pleasant, and half of the participants described avoiding the few activities available to them due to the burdens of following precautions or fear of contracting SARS-CoV-2. Anne explained, ‘I don’t care to go out too much. It’s just the plain nuisance of having to decontaminate everything when you come back in’. In T2, seven participants had challenges resuming in-person interactions with precautions. Alice described. ‘It’s that sort of hyper vigilance. You can’t just relax’. Additionally, during the relaxation of restrictions and reopening of businesses in T2 and T3, all of the participants continued to avoid reopened public spaces due to fear of contracting SARS-CoV-2 or the burden of restrictions. For some, lack of clear regulations, such as mandated masks, contributed to this. Anne, who needed to take the bus to get to medical appointments described, ‘The bus was quite crowded, and it is a bit concerning that nobody’s insisting that they wear masks’. However, by T2, five participants also expressed feeling more habituated to following pandemic restrictions. Terry described, ‘We’ve got all the protocols down, about the distancing, about the mask-wearing, about keeping to yourself, about staying at home’.

*Missing in-person engagements:* ‘The social joy pieces are totally sporadic now’ (Margret, T1). Throughout all timepoints, all participants struggled with the loss of in-person social connections. Frank shared, ‘we miss seeing our kids and grandkids’. Participants missed in-person community activities, despite the emergence of online ones, which compounded the loss of in-person interactions. Margret explained, ‘I sang in a choir too, a lovely choir… (I tried to) keep those going through video for a bit, but it wasn’t the same. And all those people I really like, and I haven’t seen any of them’. In T3 and T4, nine participants felt that reduced community engagement due to SARS-CoV-2 restrictions had resulted in their lives being monotonous. In T3, Alice shared, ‘there’s a certain amount of boredom… the pandemic is dragging on’. In T4, two participants felt that their social contacts had diminished and one of whom felt lonely. Frank described, ‘There was more social interaction on a day-to day basis with other people [in summer 2020]’. He went on to explain, ‘Human contact from a social point of view...it’s very low’.

*Declines in well-being:* ‘I’m feeling my age’ (Rose, T4). In T1 and T2, four participants noticed disrupted sleeping routines, seven experienced decreased concentration, four had an uptake in negative health habits, such as binge eating or drinking alcohol, and one began losing their hair, which they attributed to fear of contracting SARS-CoV-2. These participants also reported experiencing low mood or stress. While most participants no longer noticed these signs of stress by T3, three participants felt longevity of restrictions had affected their well-being in T4. Rose shared, ‘I have less energy than I used to... I think some of that is because of the COVID thing... Maybe I’m not getting stimulated enough’.

Participants struggled with the loss of in-person interactions and community activities in T1. Due to fear of contracting SARS-CoV-2 and the burden of restrictions, participants continued to limit social and community activities between T2–T4. Due to the stress caused by the COVID-19 pandemic, participants also reported an uptake in negative health habits, as well as declines in mental, cognitive and physical function.

#### *Theme 2: Adapting: ‘Whatever Happens, Happens. I’ll do my Best*’ *(Margret, T2)*

Participants adapted to challenges caused by the SARS-CoV-2 pandemic by exploring activities and routines to support their health and wellness and sustaining social and community connections. Adapting includes the subthemes supporting well-being and sustaining connections.

*Supporting well-being:* ‘It helps my mindset as well as my body’ (Lily, T2). In light of living with restrictions, participants engaged in activities to support their well-being and health. Several participants shared that they learned this strategy through life experience. Despite several participants noting their activity levels had declined, all participants adapted their exercise routines, which included walking in their neighbourhood or using online videos instead of going to gyms or community classes. This adaptation of exercise routines continued throughout the study period. In T4, Lily shared ‘[I] figure out a way to exercise every day safely…. keeps the spirits up’. Nine participants engaged in activities with the objective of supporting their mental well-being throughout all timepoints. In T1, Margret described journaling to cope with psychosomatic complaints that emerged early in the pandemic, sharing, ‘I call it writing my way out of depression. I learned quite a few years ago that when I got really down’.

Six participants attempted to maintain a routine between T1–T3; however, two participants tried to add variety to their days. In T2 Lily shared, ‘[I] plan something every day to look forward to. I didn’t have to do that before because I usually had something to look forward to’. During T1, participants adapted to the shutdown by taking up new projects. By T2, some participants had replaced some less meaningful activities with more meaningful ones. Sarah explained, ‘I went through that [cleaning] for about 2 weeks, and I thought, you know what? I don’t want to clean anymore. [Laughs.] …I’m doing more genealogy research…I’ve really stepped up my efforts in that regard’. In T4, three participants continued to adjust their routines to enable more pleasurable activities. Lily explained, ‘I was walking around the neighborhood seeing the same old crap over and over and over…It was depressing and boring’. As such, they took up swimming, describing, ‘I just love swimming. I mean, it’s just heavenly’.

*Sustaining connections:* ‘My local friends and friends from afar, we’re all still very close’ (Lily, T2). To connect and support friends, family and their community, nine participants sought to increase their technological literacy. For some, learning new technologies such as video chat involved asking for relatives’ help, while for others, it entailed choosing easy-to-use services. Five participants reported issues operating technology at various timepoints, which they needed to overcome. Anne described, ‘I finally stopped blaming myself if I’m having trouble with the website that’s badly designed because I can try another website and work it perfectly well’. Ten participants frequently used the phone rather than video chat to communicate. Sarah shared, ‘I have not been a very good texter or a very good phone person, but I’m learning’. Two participants described avoiding technological modes of communication altogether early in the pandemic in T2, and another two described reducing their use of technology in T4. Terry noted, ‘[zoom calls] don’t meet my need to be around people. I would rather talk to the produce clerk because it’s in-person’. They continued, ‘I chat up everybody who crosses my path. I find it helps’.

For the duration of the study, seven participants’ volunteering roles were curtailed due to pandemic restrictions. To maintain these roles, two participants continued to volunteer virtually, three participants donated money to organizations, and two resumed in-person volunteer activities with precautions in T2. Anne, who feared that art organizations would dissolve, donated to support them, stating, ‘All you can do is support them with money’.

Nine participants employed methods to nurture the quality of their relationships, such as allocating quality time together, doing shared activities or making gifts throughout the period under study. In T1, Rose described, ‘I make little puppet for when [my grandson and I] are singing together on Zoom’. These participants also noticed an improvement in the quality of some of their interpersonal relationships due to these strategies. Frank described their relationship with their fellow photo club members, ‘we almost feel more connected’. Although all of these participants simultaneously expressed missing in-person social interactions, none of the participants who noticed an improvement in the quality of relationships expressed feeling lonely.

To adapt to struggles encountered during the COVID-19 pandemic, participants adjusted their routines and engaged in activities to support their physical health and well-being. Additionally, participants employed a range of methods to stay connected, including increasing technological literacy and fostering high-quality interactions.

#### *Theme 3: Appreciating: ‘Enjoy What you Have*’ *(Frank, T3)*

During the period under study, participants appreciated the daily experiences still available to them. The theme appreciating includes the subthemes, life circumstance and experientially enjoying activities.

*Life circumstance*: ‘We have a place to live that’s secure and safe’ (Eric, T3). Participants appreciated various aspects of their life circumstance which enabled them to engage in activities to support their well-being and quality of life during the pandemic, such as their living arrangements, financial stability, geographic location and stage in life, throughout all timepoints. Eleven participants felt that being retired resulted in less financial hardship and routine disruption. In T2 Terry shared, ‘there’s no pressure to go to work….so it’s easier to form a routine’. Sarah expressed, ‘we’re retired and there’s money in the bank so, life is pretty good’. However, one participant, who was recently retired highlighted that pandemic restriction made adapting to retirement more challenging. Alice shared in T3, ‘[the COVID-19 pandemic] happened around the same time that created this sort of…strangeness for me’.

Eight participants expressed gratitude for their geographical location and home environment. In T2, Sarah shared, ‘we have a house with a yard…. We live in a neighbourhood where we can go for a walk and it’s gorgeous’. However, two participants, who lived in central urban locations, had challenges taking their daily walks due to busy sidewalks and instances of people not obeying physical distancing. Participants also appreciated circumstantial factors that enabled them to sustain connections. One participant was grateful for their proximity to their grandchildren and three participants expressed gratitude for the company of their life partner in T3 and T4. Frank shared, ‘we still have each other…we’ve just sort of been lucky in life’. Eleven participants appreciated various social systems and policies in BC or Canada. Rose expressed, ‘we are so lucky with the health care system here [in Canada]’.

*Experientially enjoying activities*: ‘Slowing down and appreciating what we have’ (Rose, T1). Throughout the pandemic, all of the participants described valuing the activities still available to them. Frank expressed, ‘we enjoy being able to get out for a walk because we’re still allowed to’. Furthermore, all participants also appreciated time freed up during the pandemic, which was utilized to pursue personal interests. Sarah, who was an avid gardener, expressed, ‘I am much more in tune with my garden now because I’m doing it regularly. It’s probably more enjoyable because of that’. Nine participants made the most of unique experiences available during each phase of the COVID-19 pandemic. During the initial onset, participants took pleasure in the stillness of the shutdown. In T1, Anne shared, ‘I really love the absence of airplanes, excess noise, excess pollution’. During the relaxation of restrictions in the summer, three older adults relished reuniting with family. Rose describes meeting her family outside for the first time, ‘[it was] just great to see them and great to have a conversation’. Eight participants also appreciated the resumption of numerous services. Anne explained, ‘the exciting thing now is that my library branch finally got in touch by email yesterday and said, “You can have your books!”’ Participants embraced the summer weather during T2 and T3 and the activities it enabled. Alice lived by the ocean and shared, ‘It’s pretty lovely… the water has been amazingly warm…I try and go every day’.

During all timepoints, participants appreciated the day-to-day activities still available to them during the COVID-19 pandemic. Participants were also grateful for living circumstances such as owning a home, living close to family, financial stability and experiencing less routine disruption, which improved their experiences during the COVID-19 pandemic.

## Discussion

This study investigated the transitions and trajectories of older adults during a 10-month period during the COVID-19 pandemic. The three themes, struggling, adapting and appreciating, summarize the range of experiences reported in this group of community dwelling older adults living in BC, Canada.

Older adults in our study struggled with reduced social interactions and reported difficulty remaining active and getting to medical appointments, as found in prior studies during the COVID-19 pandemic (Berg-Weger & Morley, 2020; Brooke & Clark, 2020). Older adults in our study felt the longevity of pandemic restrictions contributed to declines in their health and well-being, supporting studies suggesting that reductions in social interactions and exercise were associated with declines in cognitive function and well-being during the COVID-19 pandemic (De Pue et al., 2021; Noguchi et al., 2021). One barrier to older adults’ engagement in essential activities for health and wellness is that they felt uncomfortable using community spaces due to fear that the public would not abide by pandemic measures, such as social distancing, as found in prior studies (Brooke & Clark, 2020; Fiocco et al., 2021). In our study, participants revealed that when preventative measures were suggestions rather than regulations, such as those pertaining to wearing a mask indoors at some points during the study period, this discomfort was exacerbated and resulted in them avoiding those community spaces. As such, older adults’ trajectories, notably their behaviours and health, appear to have been influenced by a lack of mandated pandemic measures. This suggests that public safety measures should be mandated rather than suggested in public spaces in increase older adults’ comfort in engaging in community activities.

Older adults in our study highlighted facets of their living circumstances influenced their health trajectories during the COVID-19 pandemic. Notably, participants illuminated how access to outdoor space, as well as proximity to nature enabled them to maintain activities for physical health and well-being, supporting studies during the COVID-19 pandemic (Fiocco et al., 2021; Huntley & Bratt, 2022). Furthermore, as found by Fiocco et al. (2021), older adults in this study shared that being retired resulted in more financial stability and less routine disruption, therefore, better enabling them to continue engaging in valued activities. This highlights that publicly funded community services should be available for older adults who do not have the resources to engage in physical activities and personal interests.

Many older adults in this study sought to increase their comfort using technology to better participate in social roles and connect with individuals outside their household, echoing findings from prior studies during the COVID-19 pandemic (Haase et al., 2021; Lee et al., 2021). However, for several older adults in our study, dissatisfaction with technological substitutes shaped older adults’ transitions during the pandemic by reducing their engagement in former roles and responsibilities, such as attending community groups. Corroborating Haase et al., (2021), we found that contextual factors, namely, support from relatives and user-friendly websites and services facilitated older adults’ abilities to learn to use new technologies. However, all older adults in this study required a certain degree of computer literacy to participate, and thus, these findings may not generalize to older adults who did not meet the eligibility criteria. Many older adults in this study opted to use the phone rather than video chat to communicate with others, which (Gorenko et al., 2021) suggested may be a preferable option for delivering health care interventions for older adults with lower technology literacy. As such, to enable older adults to access essential services and participate in societal roles, health care providers and community organizations should invest in user-friendly websites in addition to maintaining telephone or in-person services. The government could also develop programs to support older adults to improve their digital literacy.

As found in prior studies, participants in this study adjusted their routines during the COVID-19 pandemic to engage in activities that were meaningful to them (Huntley & Bratt, 2022), that supported their mental and physical health, such as exercise (Ejiri et al., 2021; Takashima, Onishi & Hirano, 2020; Whitehead & Torossian, 2021), shaping their trajectories during the pandemic. Similar to prior studies during the COVID-19 pandemic, participants described their enhanced enjoyment of daily activities (Fiocco et al., 2021; Huntley & Bratt, 2022). Older adults also employed strategies to facilitate high-quality interactions, including doing shared activities or using humour, which improves social connectedness and is associated with better health (Fingerman et al., 2021; Rossignac-Milon & Higgins, 2018; Zhaoayang et al., 2019). Fiocco et al., (2021) found that older adults drew on their experiences overcoming negative life events in order to enact strategies to cope with the COVID-19 pandemic, such as taking pleasure in daily activities. We had similar findings, with participants strategically using activity to support well-being. Drawing on life course theory, older adults leveraged personal experiences to enable meaningful engagement in activities essential for health and wellness, facilitating positive transition (Bernardi et al., 2019). Given the benefits of fostering high-quality interactions and maintaining personal interests, governments should invest in services that provide older adults the opportunity to socialize and engage in meaningful activities to support their well-during longstanding global crises.

### Strength and Limitations

The majority of participants in this study were middle class and all had sufficient technological skills to participate; therefore, their experiences may not reflect those of lower socioeconomic status or individuals with lower digital literacy. Furthermore, the experiences described are limited to the British Columbian context and COVID-19 pandemic restrictions. Member checking was not completed to enrich the theme development. Additionally, there were only 12 participants in this study, limiting the ability to generalize this data to other older adults, particularly outside of BC. The initial interviews were conducted when SARS-CoV-2 case numbers were low in BC. Although the final interview was conducted once a vaccine had been developed, it was during the biggest surge in cases in BC to that point. As such, had this study occurred in a region with a different pattern of cases, alternate courses of transition may have occurred.

A strength of this study is that by adopting a longitudinal and life course perspective, factors contributing to variable experiences among older adults during the pandemic are highlighted. Furthermore, this study can be used to guide services to promote activities imperative to health and well-being for community dwelling older adults.

### Future Research

Given the fluctuating experiences described here and sample characteristics, research is needed on the long-term impact of pandemic restrictions on older adults of lower socioeconomic status, those with lower digital literacy, and racialized groups. Research evaluating programs, policies and services that can promote safe, high-quality, and in-person interactions and community activities during longstanding global crises is warranted.

## Conclusion

Guided by life course theory, this study identified three themes of struggling, adapting and appreciating. This research supports that during periods of transition, such as longstanding global crises, continued engagement in meaningful activities can support well-being, and long-term health outcomes. Due to discomfort using community spaces, older adults reduced their engagement in community activities. As such, it is imperative that local and provincial governments, as well as privately funded community organizations, implement policies and establish services that enable older adults to safely participate in community activities. Finally, this study suggests older adults can leverage their life experiences to weather periods of transition.

## Supplemental Material

sj-pdf-1-jag-10.1177_07334648221105062 – Supplemental Material for ‘Make the Most of the Situation’. Older Adults’ Experiences during COVID-19: A Longitudinal, Qualitative Study Click here for additional data file. Supplemental Material, sj-pdf-1-jag-10.1177_07334648221105062 for ‘Make the Most of the Situation’. Older Adults’ Experiences during COVID-19: A Longitudinal, Qualitative Study by E. Brooks, S. Mohammadi, W. B. Mortenson, C. L. Backman, C. Tsukura, I. Rash, J. Chan and W. C. Miller in Journal of Applied Gerontology
