# Supplementary material for: ‘Make the Most of the Situation’. Older Adults’ Experiences during COVID-19: A Longitudinal, Qualitative Study
Source: J Appl Gerontol. 2022 Jun 5;41(10):2205–13. doi: 10.1177/07334648221105062 (PMC9177809; doi:10.1177/07334648221105062)
Supplement: sj-pdf-1-jag-10.1177_07334648221105062 – Supplemental Material for ‘Make the Most of the Situation’. Older Adults’ Experiences during COVID-19: A Longitudinal, Qualitative Study [file sj-pdf-1-jag-10.1177_07334648221105062.pdf]

Appendix 1. Interview questions for timepoint one (T1).

1. **What has your experience of COVID been like?**

   Prompt: Emotionally, physically (exercise), socially.

2. **What was a typical day like for you before the COVID quarantine?**

   Prompt: What does a typical day look like for you now?

   Prompt: What is the most meaningful part of your day?

   Prompt: Which of these changes do you feel are positive?

   Prompt: Which of the changes do you feel are negative?

3. **What are you doing more of?**
4. **What are you doing less of?**
5. **What activities are you doing to connect with others?**

   Prompt: How would you describe your living situation?

6. **What activities are you doing to contribute?**

   Prompt: To your personal well-being, to society, to the world?

7. **What type of restorative activities are you engaging in?**

   Prompt: How do you feel when you are doing your daily activities?

8. **How, if at all, has the meaning of the activities you do changes with the advent of COVID?**
9. **What are some of the biggest challenges you have encountered?**
10. **What are some of the strategies and supports you have used to overcome them?**
11. **Knowing the ways you've adjusted to the current situation, what would you recommend to others?**

12. What ideas do you have about innovations that could facilitate that change?

Prompt 1. How, if it all, has your use of social media changed during this time?

Prompt 2: Describe your use of technology currently.

13. What are your future plans?

Prompt: Today, this week, monthly, before next interview, for the year
